# The complete chloroplast genome of *Tradescantia pallida* (Rose) D.R.Hunt

**DOI:** 10.1080/23802359.2020.1787262

**Published:** 2020-07-24

**Authors:** Jing Gao, Mingying Zhang, Sijing Guan, Ying Chen, Aping Liu, Yonggang Yan, Nan Wang, Gang Zhang

**Affiliations:** a College of Pharmacy, Shaanxi Qinling Application Development and Engineering Center of Chinese Herbal Medicine, Shaanxi University of Chinese Medicine, Xi’an, China; b Shaanxi Collaborative Innovation Center of Chinese Medicinal Resources Industrialization, Shaanxi University of Chinese Medicine, Xianyang, China

**Keywords:** Chloroplast genome, phylogeny, Commelinaceae

## Abstract

In this study, the complete chloroplast genome of *Tradescantia pallida* (Rose) D.R.Hunt was investigated. The whole chloroplast genome sequence is 166,086 bp in length, which consists of a 94,376 bp large single copy (LSC) and an 18,678 bp small single copy (SSC) regions, separated by a pair of 26,518 bp inverted repeat (IR) regions. The chloroplast genome of *T. pallida* encodes 131 annotated known unique genes including 86 protein-coding genes, 37 tRNA genes, and 8 rRNA genes. Phylogenetic analysis based on chloroplast genome sequences demonstrated that *T. pallida* is most closely related to *Belosynapsis ciliate*.

*Tradescantia pallida* (Rose) D.R.Hunt is a material for plant morphology and physiology study, which mainly distribute in tropical countries. The staminal hairs of *T. pallida* played an important role in studying cell-to-cell passage of small molecules (Tucker and Boss 1996). The flower of *T. pallida* can be used to detect radiation. In addition, *T. pallida* is also a good material for observing thick-angled tissues, thin-walled tissues, threaded vessel, cytoplasmic flow and posterior inclusion crystal (Crispim et al. 2012). In addition, the whole herb of *T. pallida* can be used as traditional medicines, which can activate blood, resolve stasis and remove toxin for detumescence (Dash et al. 2020), and has a significant effect on the treatment of snake bites, bruises, swelling, and rheumatism. However, there are few reports about the complete chloroplast genome of *T. pallida*.

The fresh leaves of *T. pallida* were collected from Xianyang (Shaanxi, China; 34°19’N, 108°44’E). Voucher specimen (610402191202001LY) was deposited in the herbarium of traditional Chinese Medicine, Shaanxi University of Chinese Medicine. Fresh leaves were silica-dried and taken to the laboratory. Whole genome sequencing was performed using Illumina Hiseq X10 platform (Illumina, San Diego, CA). Sequencing platform based on Sequencing By Synthesis (SBS) technology. A total of 47,670,410 clean reads were produced. The whole chloroplast genome was assembled using SPAdes v3.11.1 (http://cab.spbu.ru/software/spades/) (Bankevich et al. 2012) and then annotated by CpGAVAS and DOGMA (http://dogma.ccbb.utexas.edu/) (Liu et al. 2012). A physical map of the chloroplast genome was generated by OGDRAW.

The complete chloroplast genome of *T. pallida* (GenBank accession number: MT527961) is 166,086 bp in length, containing a large single-copy region (LSC) of 94,376 bp, a small single-copy region (SSC) of 18,678 bp, and two inverted repeat (IR) regions of 26,518 bp, and the GC content is 35.15%. It contains 86 protein-coding genes, 37 tRNA genes and 8 rRNA genes. Among which 19 genes duplicated in the IR regions, including seven protein-coding genes (rps7, rps12, rps19, rpl2, rpl23, ndhB, and ycf2), eight tRNAs (trnA-UGC, trnH-GUG, trnl-GAU, trnl-CAU, trnL-CAA, trnN-GUU, trnR-ACG, and trnV-GAC), and four rRNAs (4.5S, 5S, 16S, and 23S rRNA). In addition, 15 genes contain one intron, two genes (ycf3 and clpP) exhibit two introns.

Genome sequences were aligned using MAFFT (Yamada et al. 2016), and a maximum-likelihood phylogenetic tree including *T. pallida* and other 12 reported species (Cui and Liang 2019; Guo et al. 2019) was constructed using RAxML Version 8 with 1000 bootstrap replicates (Stamatakis 2014). The result of phylogeny analysis indicated that *T. pallida* is most closely related to *Belosynapsis ciliate* from the same family Commelinaceae (Figure 1). This complete chloroplast genome sequence of *T. pallida* will provide useful information for future studies in solving the phylogenetic relationships of *Commelina* species.

**Figure 1.**
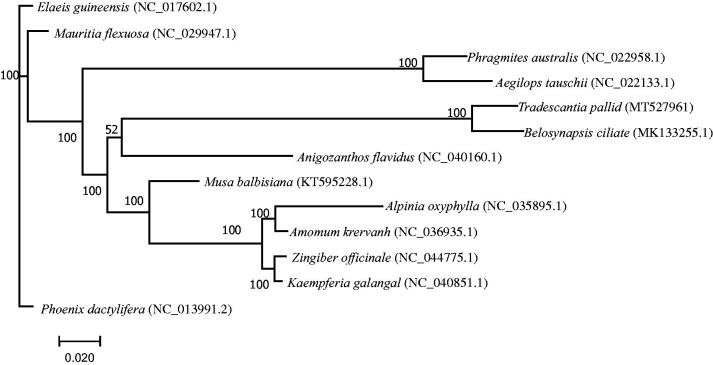
Maximum parsimony phylogenetic tree of *T. pallida* and other 12 species based on complete chloroplast genome sequences. The bootstrap support values shown next to the nodes were based on 1000 replicates.

## Data Availability

The data that support the findings of this study are openly available in National Center for Biotechnology Information (NCBI) at https://www.ncbi.nlm.nih.gov, accession number MT527961.
